# Association Between Passive Smoking and Health Among Chinese Nurses: A Cross-Sectional Study

**DOI:** 10.3389/fpubh.2021.741083

**Published:** 2021-11-11

**Authors:** Chun-ling Xia, Shi-qi Xiao, Qi-jun Wu, Xin-ying Yu, Lin-lin Xing, Li Gai, Tian-hui Xia, Hui-ling Feng, Xin-ying Zhang, Ying Guo, Yi-wei Xu, Tong-tong Fu, Xiang-hong Sun, Ling Fan

**Affiliations:** ^1^Department of Nursing, Shengjing Hospital of China Medical University, Shenyang, China; ^2^Department of Clinical Epidemiology, Shengjing Hospital of China Medical University, Shenyang, China; ^3^School of Nursing, China Medical University, Shenyang, China; ^4^School of Nursing, Hebei University of Chinese Medicine, Shijiazhuang, China

**Keywords:** passive smoking, health, nurses, CMI, second-hand smoke

## Abstract

This study aimed to investigate the association between passive smoking and physical and psychological health in Chinese nurses. Participants of this cross-sectional study comprised 2,484 non-smoking nurses. Passive smoking and demographic information were assessed using a self-administered questionnaire. Physical, psychological, and overall health status of nurses were measured using the Cornell Medical Index (CMI) health questionnaire. Multivariate-adjusted odds ratio (OR) and 95% confidence interval (CI) for nurses' health were estimated by exposure to passive smoking using unconditional logistic regression models. A total of 1,219 nurses (49.07%) were exposed to passive smoking. Of these, 609 (24.52%), 160 (6.44%), and 587 (23.63%) nurses had poorer physical, mental, and overall health, respectively. After adjusting for other confounding factors, compared with the non-passive smoking group, passive smoking was associated with poor physical (OR = 1.51, 95% CI: 1.25–1.83), mental (OR = 1.48, 95% CI: 1.07–2.07), and overall (OR = 1.58, 95% CI: 1.30–1.93) health of nurses, respectively. We also carried out subgroup analyses stratified by age, department, and professional title, which showed that most findings supported the main results. This study demonstrated that exposure to passive smoking was a risk factor for overall decreased physical and mental health status among Chinese nurses.

## Introduction

The tobacco pandemic is one of the most severe public health threats. It is well-known that subjection to second-hand smoke (SHS) has prolonged adverse health effects, and there is no safe level of exposure (1). Approximately 65.1% of non-smokers are exposed to SHS (2). Moreover, 600,000 people die annually from diseases related to SHS exposure (3), accounting for ~1.0% of global mortality (4). Previous studies have demonstrated that passive smoking is closely related to several diseases, including respiratory (5) and cardiovascular diseases (6), cancers (7), and mental disorders (8, 9). Interestingly, research on SHS exposure is increasing among women and children because of their lower smoking rates, indicating that passive smoking is a more serious public health problem than active smoking in these groups (10).

Importantly, non-smokers may be subjected to SHS in public places, the workplace, or in their home. Many countries have issued smoke-free policies to help reduce SHS exposure in public places and workplaces, but not in the home (11). Because nurses work in hospitals, which are smoke-free environments, they are an appropriate target group to examine the relationship between exposure to SHS in non-workplace environments and health. Moreover, nurses can respond accurately to technically worded questionnaires because of their health knowledge and their ability to provide comprehensive information regarding various diseases.

However, few studies have investigated the association between SHS and sub-health, defined as a decline in vitality, physiological function, and capacity for adaptation, without the presence of clinical or sub-clinical disease (12). Importantly, the Cornell Medical Index (CMI) (Chinese version) is a self-administered form with excellent reliability (Cronbach's α = 0.970), and is widely used as a sensitive indicator of health status (13, 14). Several longitudinal and cross-sectional studies have among participants of different genders, ages, and occupations (15–17).

Therefore, to our knowledge, this is the first cross-sectional study investigating the relationship between SHS and the CMI scores of non-smoking nurses. This study was conducted in a hospital in northeast China.

## Methods

### Study Design and Population

This study was a descriptive cross-sectional study conducted among a large sample of nurses. Participants were enrolled in the Health Evaluation of Occupational Nurses, which is a cohort study taking place at the Shengjing Hospital of China Medical University in Shenyang Liaoning Province, China. The cohort study was launched in 2013 to analyze the effects of genetic and environmental interactions on nurses' health.

### Data Collection

In the study, participants completed a self-administered questionnaire regarding their lifestyle and health status and donated a peripheral blood sample during a health check-up at the baseline survey every 2 years. All subjects voluntarily participated in this study and provided informed consent. This study was approved by the Ethics Committee of Shengjing Hospital of China Medical University.

The inclusion criteria were qualified nurses who underwent the annual physical examination. The exclusion criteria were nurses who were on sick leave or absent from the physical examination. A total of 2,635 participants were recruited to the present study. To examine the effect of passive smoking on health status by excluding the effect of active smoking, we limited participants to those with negligible experience of active smoking in their lifetime. Active smokers were defined as those who smoked at least one cigarette per day for more than 6 months. After excluding active smokers, there were 2,570 non-smokers left. We further excluded those missed data for SHS exposure and health status (*n* = 23). We excluded 63 male nurses in the present study. Thus, the present study included 2,484 non-smokers in the final analysis, comprising 1,219 (49.07%) passive smokers and 1,265 (50.93%) non-passive smokers.

### Exposure Assessment

The self-administered questionnaire included the following demographic characteristics and lifestyle factors: alcohol consumption, smoking status, exposure to passive smoking, education level, health status, regular diet, sleep duration and sleep quality, nursing department, employment type, working seniority, professional title, and contact time with patients. The details of this description were introduced in previous article (13).

The questionnaire examined whether the nurses were active smokers, and if so, the number of cigarettes they smoked every day. Non-smokers were asked whether they were passive smokers. Exposure level to passive smoking was assessed using a single question: “Have you inhaled the smoke of others' cigarettes (i.e., smokers) at home, in an office, or elsewhere for more than 15 minutes one day a week on average in the last year?” Responses were either “yes” or “no.”

Participants' physical and psychological health condition was measured using a modified version of the CMI health questionnaire, which comprised 18 parts and a total of 195 questions. Considering the adverse effects reported by published studies (12), subscales of the eye and ear, nervous system, respiratory system, skin, and digestive tract were used to investigate the corresponding symptoms. The questionnaire was divided into sections: A–L were related to physical disorders, and M–R referred to psychiatric/psychological morbidity. Thus, in addition to summing the total positive responses, we scored the positive responses for each of the two categories. The score range by the CMI was 0–195, and the range of the present population was 31–124, with higher scores indicating poorer levels of physical and mental health status. According to the article by Xu et al. (13), we demarcated a CMI total score of ≥40 as poor health status and M–R score of ≥20 as poor mental health. Since there was no scientific research to demark good physical health from poor conditions, we classified physical health into two categories by average: A–L score of >30 as poor physical health and A–L score of ≤ 30 as good physical health, which was often handled this way in statistics.

### Statistical Analysis

Descriptive statistics were performed to screen and compare participant characteristics between groups. Continuous variables were reported using the mean ± standard deviation, and categorical variables were represented by counts and percentages. To compare differences between groups, independent sample Student's *t*-tests were conducted for continuous variables, and the Chi-square test was used for categorical variables. Logistic regression analyses were used to calculate risk estimates to assess associations between passive smoking and health status. We first applied a model adjustment for age (years) to calculate the risk estimates of passive smoking on health. In the second regression model, we further adjusted for professional title (nurse, senior nurse, supervisor, or nursing professor); department (medical, surgical, obstetrics and gynecology, pediatrics, outpatient, other); alcohol consumption (yes or no); employment type (contract, other); regular diet (yes or no); time spent with patients (>75% or ≤ 75%); sleep duration (<6 or ≥6 h); and sleep quality (good, average, or poor). In addition, we calculated adjusted risk estimates in subgroup analyses and interaction analyses stratified by age, professional title, and department. All analyses were conducted using SPSS 21.0 (IBM, Armonk, New York, USA). All statistical tests were two-sided and statistical significance was set at *p* <0.05.

## Results

The basic characteristics of participants stratified by passive smoking are reported in Table 1. Our study included 2,484 female nurses, and 49% of them were exposed to passive smoking. Nurses who were exposed to passive smoking were younger, worked mostly in the medical and surgical departments, had lower professional titles, were likelier to consume alcohol, had irregular diets, had shorter sleep durations, and had poor sleep quality (*p* <0.05).

**Table 1:** Participant characteristics according to exposure to SHS (*n* = 2,484).

	**Total**	**Non-SHS**	**SHS**	***P*-value**
		***n* = 1,265**	***n* = 1,219**	
Age (years)				<0.05
≤ 35	1,924 (77.46)	956 (75.57)	968 (79.41)	
>35	560 (22.54)	309 (24.43)	251 (20.59)	
Working duration (years)	10.22 ± 7.27	10.64 ± 7.64	9.78 ± 6.83	<0.05
Profession title				<0.05
Nurse	729 (29.35)	339 (26.80)	390 (31.99)	
Senior nurse	1,417 (57.04)	729 (57.63)	688 (56.44)	
Supervisor or nursing professor	338 (13.61)	197 (15.57)	141 (11.57)	
Department				<0.05
Medical	651 (26.21)	304 (24.03)	347 (28.46)	
Surgical	523 (21.05)	251 (19.84)	272 (22.31)	
Obstetrics and gynecology	331 (13.33)	166 (13.12)	165 (13.54)	
Pediatrics	288 (11.59)	157 (12.41)	131 (10.75)	
Outpatient	353 (14.21)	201 (15.89)	152 (12.47)	
Other	338 (13.61)	186 (14.71)	152 (12.47)	
Alcohol consumption				<0.05
No	2,306 (92.83)	1,189 (93.99)	1,117 (91.63)	
Yes	178 (7.17)	76 (6.01)	102 (8.37)	
Employment type				<0.05
Contract	2,048 (82.45)	1,010 (79.84)	1,038 (85.15)	
Others	436 (17.55)	255 (20.16)	181 (14.85)	
Regular diet				<0.05
Yes	1,544 (62.16)	825 (65.22)	719 (58.98)	
No	940 (37.84)	440 (34.78)	500 (41.02)	
Time spent with patients				<0.05
>75%	1,669 (67.19)	875 (69.17)	794 (65.14)	
≤ 75%	815 (32.81)	390 (30.83)	425 (34.86)	
Sleep duration (hours)				<0.05
<6	920 (37.04)	439 (34.70)	481 (39.46)	
≥6	1,564 (62.96)	826 (65.30)	738 (60.54)	
Sleep quality				<0.05
Good	1,048 (42.19)	574 (45.37)	474 (38.88)	
Average	1,059 (42.63)	521 (41.19)	538 (44.14)	
Poor	377 (15.18)	170 (13.44)	207 (16.98)	

*SHS, second-hand smoke*.

Table 2 shows that nurses exposed to passive smoking had poor overall health than the non-exposed group (28.14 and 19.29%, respectively, *p* < 0.05), and significant declines in physical (28.88 and 20.32%, respectively, *p* < 0.05) and mental health (7.71 and 5.22%, respectively, *p* < 0.05).

**Table 2:** Association between SHS and health status.

	**Total**	**Non-SHS**	**SHS**	***P*-value**
		***n* = 1,265**	***n* = 1,219**	
Physical health				<0.05
A-L score ≤ 30	1,875 (75.48)	1,008 (79.68)	867 (71.12)	
A-L score > 30	609 (24.52)	257 (20.32)	352 (28.88)	
Mental health				<0.05
M-R score <20	2,324 (93.56)	1,199 (94.78)	1,125 (92.29)	
M-R score ≥ 20	160 (6.44)	66 (5.22)	94 (7.71)	
CMI				<0.05
Total score <40	1,897 (76.37)	1,021 (80.71)	876 (71.86)	
Total score ≥ 40	587 (23.63)	244 (19.29)	343 (28.14)	

*SHS, second-hand smoke*.

Nurses' exposure to passive smoking was associated with health status risk (see Table 3). Specifically, nurses who were exposed to passive smoking had poor physical, mental, and overall health. The power of our study was 0.9977 after calculation.

**Table 3:** ORs and 95% CIs for nurses' health according to exposure to SHS among non-smokers.

	**Non-SHS**	**SHS**
	***n* = 1,265**	***n* = 1,219**
Physical health		
Age-adjusted OR (95% CI)	Ref.	1.59 (1.32–1.91)
Multivariate-adjusted OR^*^(95% CI)	Ref.	1.51 (1.25–1.83)
Mental health		
Age-adjusted OR (95% CI)	Ref.	1.51 (1.09–2.09)
Multivariate-adjusted OR^*^(95% CI)	Ref.	1.48 (1.07–2.07)
CMI		
Age-adjusted OR (95% CI)	Ref.	1.63 (1.35–1.97)
Multivariate-adjusted OR*(95% CI)	Ref.	1.58 (1.30–1.93)

*SHS, second-hand smoke; CI, confidence interval; OR, odds ratio.*

* *Adjusted for age (categorical variable), profession title, department, alcohol consumption, employment type, regular diet, time spent with patients, sleep duration, and sleep quality*.

Subgroup analyses stratified by age, professional title, and department (Table 4) showed that the direction of most findings were consistent with the main results; however, not all results showed statistical significance.

**Table 4:** Subgroup and interaction analysis.

	**Physical health**			**Mental health**			**CMI**		
	**Non-SHS**	**SHS**	p^*^	**Non-SHS**	**SHS**	p^*^	**Non-SHS**	**SHS**	p^*^
Age (years)			0.86			0.93			0.19
≤ 35	Ref.	1.52 (1.23–1.90)		Ref.	1.50 (1.03–2.17)		Ref.	1.73 (1.38–2.16)	
>35	Ref.	1.47 (0.97–2.24)		Ref.	1.31 (0.60–2.86)		Ref.	1.13 (0.74–1.75)	
Profession title			0.39			0.40			0.04
Nurse	Ref.	1.62 (1.13–2.33)		Ref.	1.96 (0.98–3.91)		Ref.	2.28 (1.56–3.33)	
Senior nurse	Ref.	1.56 (1.21–2.01)		Ref.	1.44 (0.94–2.19)		Ref.	1.45 (1.12–1.87)	
Supervisor or nursing professor	Ref.	1.04 (0.58–1.85)		Ref.	0.86 (0.32–2.36)		Ref.	1.06 (0.59–1.91)	
Department			0.75			0.86			0.19
Medical	Ref.	1.87 (1.27–2.76)		Ref.	1.08 (0.57–2.07)		Ref.	2.13 (1.44–3.14)	
Surgical	Ref.	1.37 (0.91–2.07)		Ref.	1.67 (0.83–3.37)		Ref.	1.77 (1.16–2.70)	
Obstetrics and gynecology	Ref.	1.27 (0.68–2.39)		Ref.	1.07 (0.28–4.15)		Ref.	0.81 (0.40–1.62)	
Pediatrics	Ref.	1.51 (0.84–2.72)		Ref.	1.69 (0.49–5.81)		Ref.	1.65 (0.89–3.07)	
Outpatient	Ref.	1.35 (0.83–2.20)		Ref.	2.21 (1.00–4.88)		Ref.	1.13 (0.69–1.88)	
Other	Ref.	1.52 (0.90–2.55)		Ref.	1.52 (0.68–3.39)		Ref.	1.70 (1.02–2.84)	

*SHS, second-hand smoke.*

* *p for interaction*.

## Discussion

### Main Findings

To our knowledge, this is the first study to investigate passive smoking and nurses' health using CMI as a measurement indicator among northeastern Chinese female nurses. We found a significant association between passive smoking and nurses' health. Specifically, exposure to passive smoking deteriorated nurses' physical, mental, and overall health. The results of subgroup analysis also supported the main findings.

### Comparison With Other Studies

Our results suggest that exposure to SHS is related to physical and mental health, which is consistent with previous studies (18–21). For example, a study on environmental tobacco smoke (ETS) in China indicated that the estimated acute health risks after 1–2 h of ETS exposure would be 29% (20). Moreover, a meta-analysis of 11 cross-sectional studies demonstrated that exposure to SHS among non-smokers was significantly associated with depressive symptoms and psychological distress (21). A South Korean study investigated the lifetime costs and health outcomes of adult women exposed to SHS at home, and found that women exposed to SHS had higher health expenditures and lower life expectancies (22). In addition, health status is related to the amount of SHS. Specifically, women who live with smokers have poorer health than women who do not, and the more smokers they live with, the worse their health (23). However, our findings were inconsistent with several studies (24, 25). For example, a UK national population-based prospective cohort study demonstrated that there was a non-significant association between objectively measured SHS exposure and poor mental health (24). This can be explained from a cultural perspective. That is, traditional Chinese Confucianism advocates a modest and harmonious approach, which prevents people from directly expressing their emotions. Thus, when individuals encounter unpleasant situations such as exposure to SHS, most opt to endure the circumstances, rather than prevent it (26). Variations in research objects and mental health assessment methods may also explain the contradictory evidence. Thus, it is necessary to conduct further research in this field.

### Potential Biological Mechanisms

SHS is a complex reactive mixture containing over 4,700 chemicals, including hazardous amines, carbonyls, hydrocarbons, and metals (27, 28). Moreover, mainstream tobacco smoke contains 11 strains of human carcinogens which can cause various types of cancer (29). Nicotine is the main component of tobacco and is a sympathetic stimulant that prompts the release of catecholamines and other neurotransmitters (30, 31). Cotinine, which is the main product of nicotine after primary metabolism, is associated with psychological distress and the risk of future mental illness (32). Thus, exposure to SHS can increase the levels of corticotropin releasing hormone and adrenocorticotropic hormone, and gradually affects individuals' emotions and cognitions (33–35). Previous studies indicate that passive smoking is related to endocrine and metabolic changes (36–39), which may lead to sub-health status.

### Strengths and Limitations

Our study has several strengths. First, this is the first study investigating nurses' health and passive smoking using CMI as a measurement indicator. Second, this study has a large sample size and can be used for subgroup analysis. Third, the inclusion of nurses as study participants can reduce measurement error, because their health knowledge and abilities can provide comprehensive and accurate information. Finally, we performed a subgroup analysis of passive smoking, and the results supported our main findings.

However, our study has several limitations. First, this is a cross-sectional study which cannot determine the causal relationship between passive smoking and health status. Second, the exposure to passive smoking was self-reported. We did not measure biomarkers (such as cotinine levels); thus, the actual prevalence of SHS exposure may have been underestimated. Moreover, we did not investigate the amount of passive smoking; therefore, we could not further explain the relationship between the dose of passive smoking and nurses' health. In addition, in other studies, passive smoking environments were divided into public places, workplaces, and in the home (6). In our study, the subjects were nurses who worked in a smoke-free hospital, who were exposed to SHS both in public places and at home. However, passive smoking in public places and in the home could not be separated. Finally, passive smoking is related to many factors, such as gender, age, alcohol consumption, lifestyle, socio-economic status, and allergic sensitization. In our study, we adjusted for age, duration of service, professional title, department, alcohol consumption, employment type, diet, contact time with patients, sleep duration and sleep quality. Nevertheless, some potential confounding factors remain, but may have little impact on our results. Although this study has a large sample size and can be used for subgroup analysis, not all results showed statistical significance which may be attributed to limited sample sizes in specific subgroups. Further studies are warranted to pay more attention to solve this issue in the future.

### Conclusions and Interpretations

In conclusion, our study provides novel evidence for the relationship between SHS exposure and health among female nurses in China. Exposure to SHS is associated with overall, physical, and mental health.

## Data Availability Statement

The raw data supporting the conclusions of this article will be made available by the authors, without undue reservation.

## Ethics Statement

The studies involving human participants were reviewed and approved by Shengjing Hospital of China Medical University. The patients/participants provided their written informed consent to participate in this study.

## Author Contributions

LF, S-qX, X-hS, and C-lX: conceptualization and funding acquisition. T-hX, Q-jW, and H-lF: contributed materials/analysis tools. T-tF, YG, L-lX, X-yY, and LG: helped in data collection and field operations. All authors helped prepare the manuscript and approved the submitted version.

## Funding

This project was funded by the Department of Science and Technology of Liaoning Province (Grant Number 2018225005).

## Conflict of Interest

The authors declare that the research was conducted in the absence of any commercial or financial relationships that could be construed as a potential conflict of interest.

## Publisher's Note

All claims expressed in this article are solely those of the authors and do not necessarily represent those of their affiliated organizations, or those of the publisher, the editors and the reviewers. Any product that may be evaluated in this article, or claim that may be made by its manufacturer, is not guaranteed or endorsed by the publisher.
